# Modeling and estimating the feedback mechanisms among depression, rumination, and stressors in adolescents

**DOI:** 10.1371/journal.pone.0204389

**Published:** 2018-09-27

**Authors:** Niyousha Hosseinichimeh, Andrea K. Wittenborn, Jennifer Rick, Mohammad S. Jalali, Hazhir Rahmandad

**Affiliations:** 1 Grado Department of Industrial and Systems Engineering, Virginia Tech, Blacksburg, VA, United States of America; 2 Department of Human Development and Family Studies, Michigan State University, East Lansing, MI, United States of America; 3 Division of Psychiatry and Behavioral Medicine, College of Medicine, Michigan State University, Grand Rapids, MI, United States of America; 4 Sloan School of Management, Massachusetts Institute of Technology, Cambridge, MA, United States of America; Public Library of Science, UNITED KINGDOM

## Abstract

The systemic interactions among depressive symptoms, rumination, and stress are important to understanding depression but have not yet been quantified. In this article, we present a system dynamics simulation model of depression that captures the reciprocal relationships among stressors, rumination, and depression. Building on the response styles theory, this model formalizes three interdependent mechanisms: 1) Rumination contributes to ‘keeping stressors alive’; 2) Rumination has a direct impact on depressive symptoms; and 3) Both ‘stressors kept alive’ and current depressive symptoms contribute to rumination. The strength of these mechanisms is estimated using data from 661 adolescents (353 girls and 308 boys) from two middle schools (grades 6–8). These estimates indicate that rumination contributes to depression by keeping stressors ‘alive’—and the individual activated—even after the stressor has ended. This mechanism is stronger among girls than boys, increasing their vulnerability to a rumination reinforcing loop. Different profiles of depression emerge over time depending on initial levels of depressive symptoms, rumination, and stressors as well as the occurrence rate for stressors; levels of rumination and occurrence of stressors are stronger contributors to long-term depression. Our systems model is a steppingstone towards a more comprehensive understanding of depression in which reinforcing feedback mechanisms play a significant role. Future research is needed to expand this simulation model to incorporate other drivers of depression and provide a more holistic tool for studying depression.

## Background

Major depressive disorder is a systemic syndrome [1]. Previous research has identified multiple interactions among depressive symptoms, rumination, and stressful events. Response styles theory [2, 3] defines rumination as repetitively and passively focusing on symptoms of distress and the causes and consequences of the symptoms without actively engaging in coping or problem-solving. Rumination is a robust predictor of the onset of depression in children [4], adolescents [5], and adults [6]. Stress caused by difficult life events increases an individual’s vulnerability to rumination and depression [5, 7]. On the other hand, multiple studies have indicated that the interaction of stress and rumination predicts both onset and severity of depression [8–11]. In addition, recent studies have found that rumination moderates the association between stress and depression [5, 12, 13]. While research supports a link between stress and rumination, less is known about how these processes unfold over time and how their interactions contribute to depression.

Ruscio and colleagues suggested three potential pathways for the link between rumination, stress, and depression [14]. First, rumination after stressful events may lead to the negative affect that is an important feature of mood disorders. Second, rumination may cause dysfunctional behaviors such as reduced activity or withdrawal that worsen depressive symptoms. Third, rumination might worsen the effects of past stressors by “keeping the stressor ‘alive’—and the individual activated—even after the stressor has ended” [14]. Using ecological momentary assessment in which data were collected from participants multiple times per day, Ruscio and colleagues examined the first two pathways and found that rumination mediated the association between stress and depressive symptoms. They also found that individuals with major depressive disorder were more likely to ruminate in response to stressors than individuals with no psychopathology. The third pathway was not tested. Nolen-Hoeksema and colleagues examined the reciprocal relationship between rumination and depression without including stressors in the model [15]. They showed that a higher rumination score predicted elevated depressive symptoms in the next year and depressive symptoms predicted a significant increase in ruminative style over time.

Other variables can influence the relationship between rumination and depression. For instance, rumination was found to mediate the relationship between cognitive control and depressive symptoms [16]. Rumination also mediates the relation between trait anger and depression. In other words, individuals prone to experience more anger might become depressed in situations under which they tend to ruminate [17, 18]. Co-rumination, or ruminating with peers, was found to moderate the relationship between stress and depression [12]. Those with high levels of co-rumination are more likely to activate negative schemas, which in turn increases depressive symptoms [19]. Stress may contribute to depression through other pathways, including biological mechanisms such as HPA axis dysfunction [20–22] and inflammation [23, 24].

The complex feedback mechanisms among the drivers of major depressive disorder call for methods that can both estimate multiple interactions and synthesize existing evidence [25]. System dynamics provides one such methodology which has provided important insights in health research on diabetes [26, 27], heart disease [28], and post-traumatic stress disorder [29], among others. System dynamics has also been used for qualitatively capturing feedback processes underlying depression [1], but these feedback mechanisms have not been quantified.

In this study, we use system dynamics to develop a simulation model of depression based on the response styles theory [15]. We model and estimate the interactions among rumination, depressive symptoms, and stressors that have been hypothesized in prior research. While many other feedback mechanisms could be included in a comprehensive model of depression, we limit our focus to these interactions because they have strong theoretical support, and data for estimating these interactions exists. Moreover, few prior studies capture even this level of complexity, so in contributing to a cumulative science of depression we hope our study can act as a steppingstone but not as a final comprehensive model; such a comprehensive model, if ever feasible, can only come after many smaller and more modest models are developed, estimated, refined, and combined.

A novel estimation method, documented in a previous methodological article [30], was used for calibrating the model. Results inform the strength of various mechanisms and reinforcing loops relevant to depression. In addition, we estimate the mean time that a stressor is kept active (i.e., the time a person is activated by the stressor, which provides an estimate for the duration of a stressor’s impact in the absence of any intervention) for boys and girls, finding a significant asymmetry. We then simulate the model for 32 unique profiles of individuals to better understand individual variations depending on initial conditions of individuals and their exposure to stressors.

The present study contributes to the literature in several ways. First, prior studies have not estimated the endogenous relationships between stress, rumination, and depression simultaneously; instead, they have investigated the bidirectional relationship between either rumination and depression [31] or stress and depression [7, 32]. Second, this study examines the hypothesis of Ruscio and colleagues, which asserts that rumination prolongs the effects of stressful events by keeping an individual activated [14]. Our major contribution lies in capturing this untested mechanism in our dynamic model and estimating its impact in interaction with rumination and depressive symptoms. Third, this study is the first to use system dynamics to quantify feedback mechanisms involved in depression and offers a template for developing other individual-level simulation models of depression.

## Methods

A system dynamics model of interactions between depressive symptoms, rumination, and stress was developed and estimated based on data from a longitudinal study of adolescent mental health carried out by McLaughlin and Nolen-Hoeksema [33]. Below we briefly summarize the empirical data. Then, given the novelty of the use of system dynamics in this context, we provide a brief background on the approach and then discuss the simulation model and estimation approach in more detail.

### Participants

The participants were 1,057 adolescents from two middle schools (Grades 6–8) in central Connecticut. Students in the self-contained special education classrooms and technical programs who were not present in the school for the majority of the day were excluded. The racial/ethnic characteristics of the sample were: 56.9% (N = 610) Hispanic/Latino, 13.2% (N = 141) non-Hispanic White, 11.8% (N = 126) non-Hispanic Black, 9.3% (N = 100) biracial/multiracial, 2.2% (N = 24) Asian/Pacific Islander, 0.8% (N = 9) Middle Eastern, 0.2% (N = 2) Native American, and 4.2% (N = 45) other racial/ethnic groups. Based on the school records, 62.3% of the students qualified for free or reduced lunch. The per capita income of the community where the two schools were located is $18,404, which is considered a low socioeconomic status community. We excluded participants with missing values and the final sample includes 661 individuals. Twenty-eight percent of participants (N = 221) in the baseline assessment did not complete the assessment at Time 2, and 20.4% (N = 217) did not complete the assessment at Time 3. Attrition was primarily related to leaving the school district. From 2000 to 2004, 22.7% of students had left the school district [34]. Those who did not complete the second and third assessments did not differ from the participants in the first assessment in terms of grade level, race/ethnicity, or being from a single-parent household (*ps* > 0.10). However, they were more likely to be female (*χ*^2^(1) = 6.85, p < 0.01). The level of rumination and depression of those who did not complete at least one of the follow-up assessments did not differ from those who completed all assessments (*ps* > 0.10).

### Instruments

Data were collected over a span of seven months. The following instruments were used to measure stressful life events (at times 1 and 3), rumination levels (at times 1, 2 and 3), and depressive symptoms (at times 1 and 3). The time between the first and second assessments was four months, and there were three months between the second and third assessments.

#### Stressful life events

The Life Events Scale for Children [35] contains 25 examples of stressful life events. Participants were asked to indicate whether they had experienced any of the events in the past six months (e.g., “Your parents got divorced” and “You got suspended from school”). The test-retest reliability of this measure over a 2-week period is high [36, 37].

#### Rumination

The Children’s Response Styles Questionnaire (CRSQ; [4]) measures the level of rumination, distraction, and problem-solving in response to sad feelings. This measure was modeled after the Response Styles Questionnaire [2], which was developed for adults. The CRSQ is composed of 25 items that are grouped into three scales: 1) Ruminative Response subscale, 2) Distracting Response subscale, and 3) Problem-Solving subscale. In the present study, only the Ruminative Response subscale was used. For each item, children are asked to identify how often they respond a certain way when they feel sad (*almost never* = 1, *sometimes* = 2, *often* = 3, or *almost always* = 4). The Ruminative Response subscale (CRSQ-Rumination) includes 13 items and generates a score between 13 and 52. Sample items include “Think about how alone you feel,” “Think about a recent situation wishing it had gone better,” and “Think why can’t I handle things better?” Previous studies reported good reliabilities for the CRSQ-Rumination subscale [4]. In this sample, the subscale was reliable (*α* = 0.86).

#### Depressive symptoms

The Children’s Depression Inventory (CDI) includes 27 items that evaluate the presence and severity of depression in children and adolescents [38]. Each item includes three statements from which the respondents chose the one that best describes them in the past two weeks (e.g., “I am sad once in a while,” “I am sad many times,” and “I am sad all the time”). The item related to suicide was removed at the request of the human subjects committee and school officials. Adding the remaining 26 items generates a score that ranges from 0 to 52. The CDI exhibited good reliability in this sample (*α* = 0.82).

### System dynamics modeling

System dynamics is a methodological approach for understanding the structure and analyzing the dynamics of complex systems [39]. System dynamics allows for quantifying nonlinear and reciprocal influences among variables, making it an appropriate tool when endogenous feedback is important for understanding a phenomenon. In addition, system dynamics accounts for accumulations (sources of inertia) and the speed of change in states of a system. For instance, a system dynamics model of depression and rumination takes into account the reciprocal relationship (i.e., feedback loop) between the two variables such that rumination increases depressive symptoms, and more depressive symptoms lead to a higher level of rumination. In addition, system dynamics incorporates the speed of change in inertial factors. For instance, rumination does not develop overnight. It accumulates over time and its inertia influences the dynamics of depressive symptoms.

Mathematically, typical system dynamics models consist of a system of ordinary differential equations, potentially influenced by exogenous and/or random inputs, and can be simulated to track the results of different assumed model structures and parameters. Statistical estimation of these models makes it possible to identify the strength of hypothesized causal pathways based on observed data. The development of a system dynamics model is an iterative process and often involves the following steps. First, the problem that specifies the phenomenon to be explained is articulated. Second, a theory or set of dynamic hypotheses about potential causes of the problem are specified. Third, key variables, including sources of inertia (i.e., accumulation or stock variables), are identified. Fourth, a causal diagram that maps the causal relationships among the variables is developed by reviewing the literature and consulting experts and policymakers. Fifth, the model is formulated, refined, and estimated until it is robust, reliable and able to replicate historical data. The refinement process is iterative and involves various tests to build confidence in the model [40–42]. Finally, different scenarios and policies are formulated and the model is simulated to identify high-leverage policies [39].

Our motivation for using system dynamics in this research is based on three distinct benefits that this methodology can bring to the study of depression. First, depression is a complex and persistent problem, not caused by a ‘common cause,’ and as a result, a ‘causal systems perspective’ is needed to analyze the disorder [43, 44]. A system dynamics approach allows for explicitly capturing the multiple feedback mechanisms, latent variables, and accumulations relevant to understanding depression. While structural equation models and simultaneous equation estimation can partially accommodate feedback and latent variables, system dynamics models incorporate all of these and allow for potential nonlinearities among variables. Second, these models are simulated in continuous time, enabling us to separate the actual unfolding of events (which is continuous in time) from the discrete measurements used for estimation. For instance, depressive symptoms of diverse patients can be simulated continuously over time to examine their symptom trajectories under different conditions. Finally, by accommodating more realistic mechanisms and broader feedback mechanisms, these simulation models can provide input into intervention analysis and design.

### Developing and estimating a model of depression

**Fig 1:**
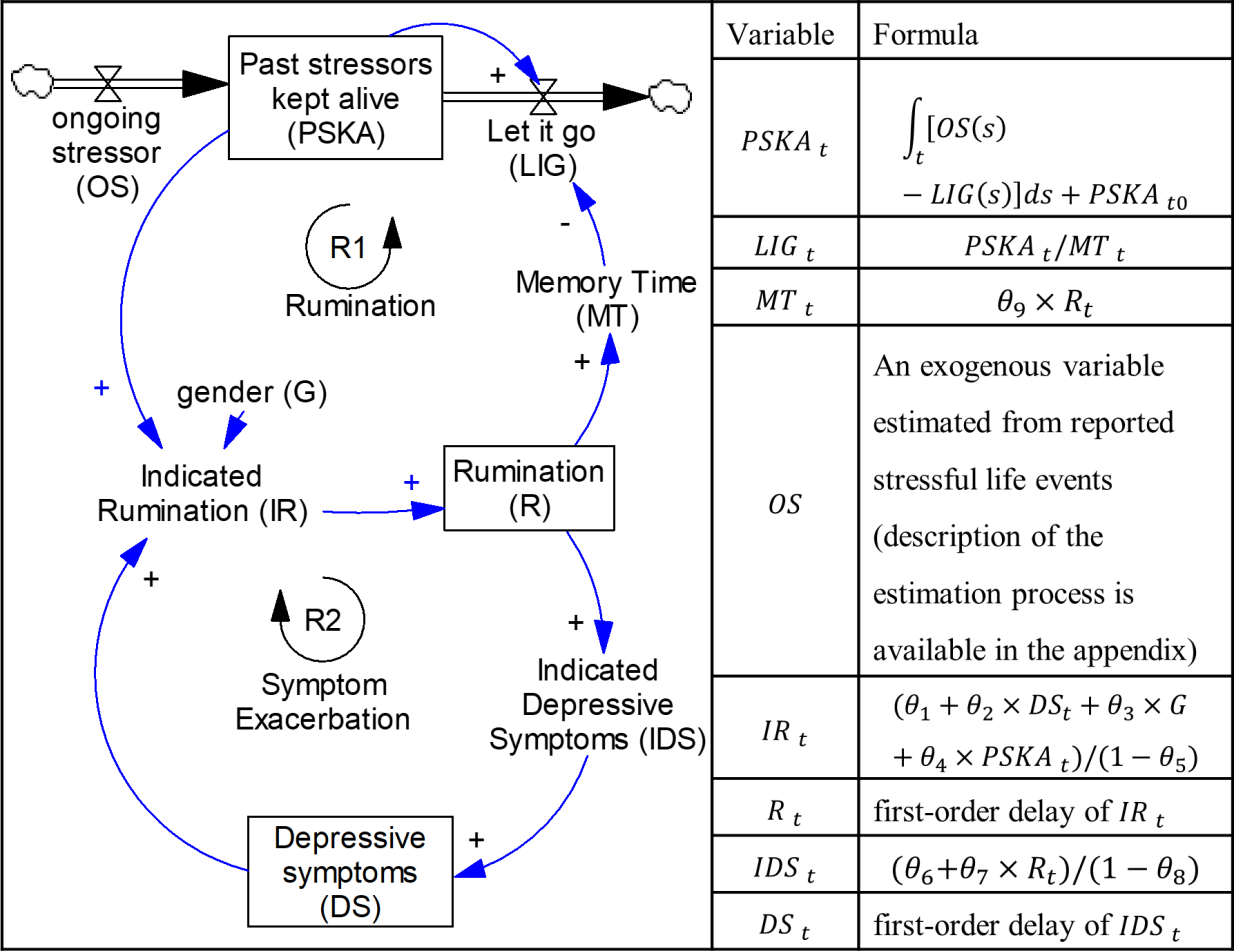
The depression-rumination conceptual model. Boxed variables represent stocks (or states), and double arrows with valves depict flows into/out of the stocks. Single-line arrows present hypothesized causal relationships—their strength is estimated below. A stock variable, which is mathematically represented as an integral, is the accumulation of the difference between its inflows and outflows.

We developed a system dynamics simulation model of depression that simultaneously captured the bidirectional relationships among depressive symptoms, rumination, and stressors at the individual level. Our model included two major feedback loops that are based on the mechanisms proposed by Ruscio and colleagues [14], depicted in reinforcing feedback loop 1 (Fig 1, loop R1: ‘Rumination’), and the response styles theory [15], illustrated in reinforcing feedback loop 2 (Fig 1, loop R2: ‘Symptom Exacerbation’). These two feedback loops are self-reinforcing such that an initial change in the loop comes back to cause a further change in the same direction—e.g., an initial increase will cause a further increase. Feedback loop R1 captures the idea that after experiencing a stressor, an individual prone to rumination spends time ruminating about the stressful events, keeping those stressors active, increasing the chances of even more rumination. In other words, rumination intensifies sensitivity to stressors by keeping a person activated and the stressor “alive” [14]. This leads to more rumination and depressive symptoms. Feedback loop R2 hypothesizes that more rumination leads to more depressive symptoms, and a higher level of depressive symptoms causes even more rumination [31]. It should be noted that feedback loops R1 and R2 are interconnected (see Fig 1). This is important because, similar to any other complex system, the interaction between the feedback loops in the model gives rise to other potential dynamics—we discuss these dynamics further in the results section.

Stressors that contribute to rumination and subsequent depression are those that happened in the past but still keep an individual activated; as a result, they are modeled as a stock variable—a boxed variable named ‘*past stressors kept alive*’ in Fig 1. A stock variable accumulates units (e.g., patients, materials, information) and mathematically is the accumulation of the difference between its inflows and outflows (represented as double arrows with valves in Fig 1). A bathtub offers a simple example of a stock variable with one inflow and one outflow. The level of water depends on the initial level of water in the tub and its net flow (i.e., the level of water in the bathtub increases when the inflow is larger than the outflow). The same argument applies to the stock of *past stressors kept alive*. The level of *past stressors kept alive* depends on the initial level of stressors (i.e., *prior stressors*) and the net flow of stressors (*past stressors kept alive* increases if *ongoing stressors* is greater than ‘*let it go’*). *Let it go* resembles the drain of the bathtub and is a function of *memory time* and *past stressors kept alive* (*let it go* equals *past stressors kept alive* divided by *memory time*). *Memory time* captures how fast an individual comes to terms with past stressors, and is formulated as the product of *rumination* and a parameter estimated in the calibration process (θ_9_, with the estimated value of 1.47). The higher the *rumination* level, the greater the *memory time* and the lower the *let it go* flow. As *let it go* declines, past stressors accumulate in the stock of *past stressors kept alive*, which increases the level of *rumination* even further (Reinforcing loop R1). Detailed formulations of the model are available in S1 File Appendix, and the results section provides more details.

This model is estimated using the indirect inference method [45, 46]. In this method, sources of stochastic environmental variations (i.e., process noise) are added to the model. The parameters of the model and the environmental factors are then estimated to match the aggregate auxiliary statistics of empirical data. Indirect inference is specifically suitable for estimating complex models with intractable likelihood functions. The online S1 File Appendix provides the details for the model formulations and the indirect inference estimation method. Additional details on the development of the simulation model and estimation method are discussed in [30]. For parameter estimation, we used the dataset of 661 adolescents collected by McLaughlin and Nolen-Hoeksema [33] that is summarized in the methods section.

## Results

Table 1 summarizes the means and standard deviations for all measures at each assessment time for all participants (*N* = 661), as well as separately for boys (*N* = 308) and girls (*N* = 353). Girls reported higher levels of rumination at all evaluation times (*p* = 0.00) and more symptoms of depression at Time 1 (*p* = 0.03) and Time 3 (*p* = 0.08). There was no gender difference in terms of experiencing stressful life events at Time 1 (*p* = 0.98) and Time 3 (*p* = 0.27).

**Table 1:** Summary of measures.

Variable	Time 1	Time 2	Time 3
Depressive symptoms (Total)	9.48 (6.28)	-	9.78 (7.64)
Depressive symptoms (Girls)	9.98 (6.45)		10.28 (7.58)
Depressive symptoms (Boys)	8.91 (6.04)		9.22 (7.68)
Rumination (Total)	11.59 (7.52)	10.85 (7.62)	9.95 (7.95)
Rumination (Girls)	12.78 (7.71)	12.24 (7.98)	11.49 (8.24)
Rumination (Boys)	10.23 (7.06)	9.25 (6.86)	8.19 (7.22)
Stressful life events (Total)	4.96 (3.32)	-	4.20 (3.70)
Stressful life events (Girls)	4.97 (3.14)		4.35 (3.48)
Stressful life events (Boys)	4.96 (3.52)		4.03 (3.93)

*Note*. Standard deviations are in parentheses.

Table 2 reports the parameters of the estimated model [30]. In interpreting the results, it is important to note that one cannot claim empirical causality for any of the discussed relationships, given the observational nature of the data. On the other hand, once a simulation model is quantified, it is possible to run synthetic controlled experiments and discuss causality within the simulation results. In the results description, the use of causal language is only intended to apply to the estimated simulation model. The gender coefficient (θ_3_) is significant, which implies that girls ruminate more than boys. The effect of stress on rumination (θ_4_) and the effect of rumination on memory time (θ_9_) are also significant, indicating that rumination increases sensitivity to stressful events by keeping the person activated and the stressor active. The effect of rumination on depression (θ_7_) is significant, while the effect of depression on rumination (θ_2_) is positive but not significant at a 95% confidence level. Overall, these results provide strong support for the Rumination reinforcing loop, moderate evidence for the Symptom Exacerbation loop, and a significant gender effect in rumination rates.

**Table 2:** Estimated parameters using indirect inference.

Unknown Parameters	Estimated Parameters
Rumination Constant (θ_1_)	-1.2504 (0.991)
Effect of Depression on Rumination (θ_2_)	0.4236 (0.301)
Gender Coefficient (θ_3_)	2.5152 (1.002)*
Effect of Stress on Rumination (θ_4_)	0.2518 (0.117)*
Rumination Coefficient (θ_5_)	0.1639 (0.495)
Depression Constant (θ_6_)	0.3730 (0.039)*
Effect of Rumination on Depression (θ_7_)	0.0699 (0.003)*
Depression Coefficient (θ_8_)	0.8894 (0.004)*
Effect of Rumination on Time Constant (θ_9_)	1.4741 (0.051)*
RumNoise Standard Deviation (θ_10_ = $\sigma_{r}^{2}$)	7.8735 (4.088)
DepNoise Standard Deviation (θ_11_ = $\sigma_{d}^{2}$)	0.0002 (0.016)
Correlation Time (θ_12_)	1.6008 (0.793)*

*Note*. Standard errors are presented in parentheses.

*statistically significant at 95% confidence level

### Average memory time for adolescents with different characteristics

**Fig 2:**
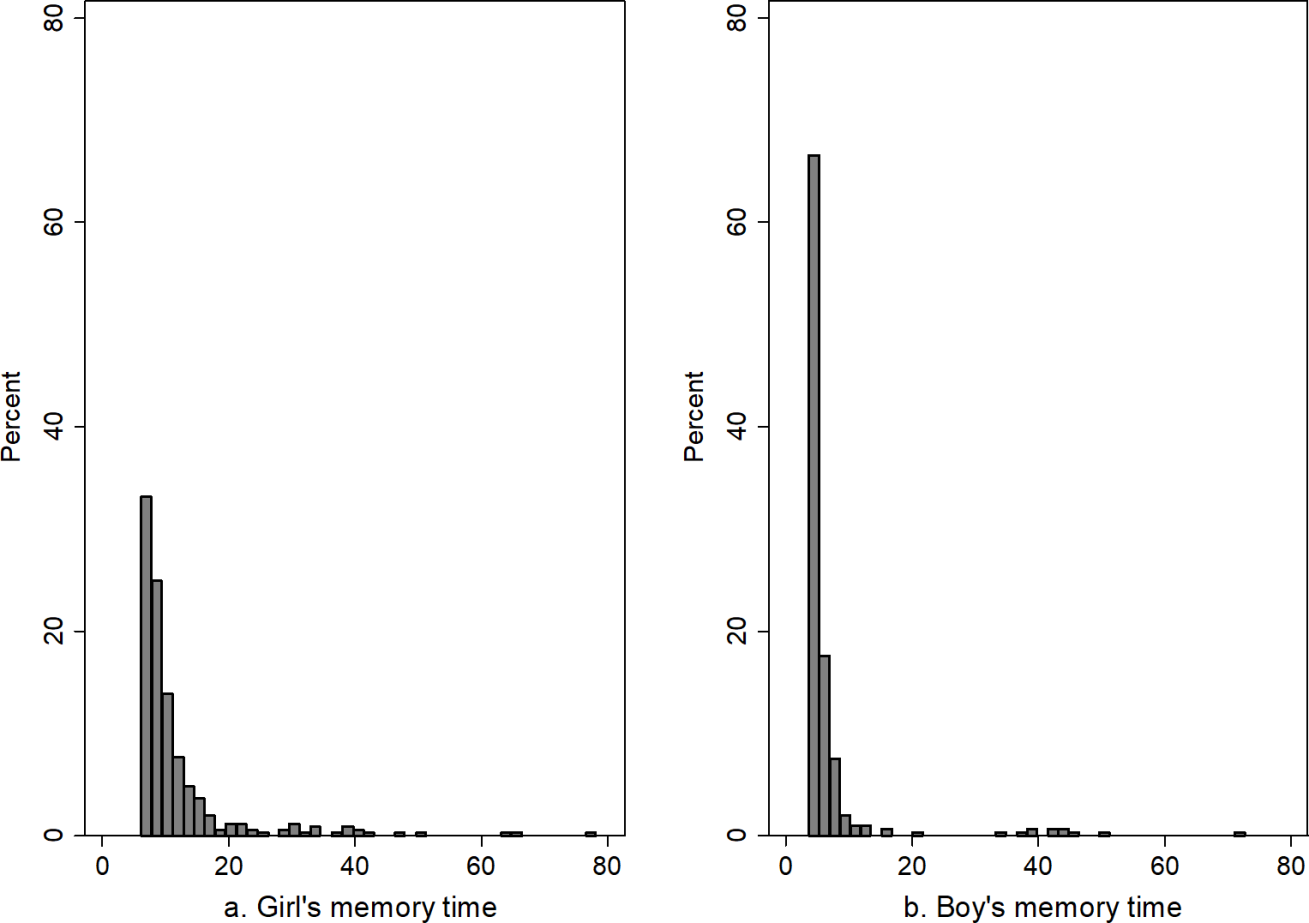
Distribution of memory time for girls and boys.

In this section, we report the average memory time (i.e., the time it takes to release a stressor) for all participants by gender (first row in Table 3) and for depressed individuals with high and low rumination by gender (second and third row in Table 3), given the estimated strength of the Rumination loop. As discussed in the methods section, memory time is defined based on the pathway hypothesized by Ruscio and colleagues, in which rumination exacerbates the effect of stressors by keeping stressors active. Memory time captures the time over which an individual releases a stressor and is proportional to rumination (i.e., memory time equals rumination multiplied by a parameter, θ_9_, with the estimated value of 1.47). The higher the *rumination* level, the greater the *memory time*, and the longer it takes the stressors to leave the stock of *past stressors kept alive* (Fig 1). We separately report average memory time of depressed patients (i.e., those with depressive symptoms above the cut-off value of 16 [47]) with high or low rumination (i.e., for individuals above/below mean rumination) because memory time is a function of rumination. A single value of θ_9_ (reported in Table 2) is estimated for all participants, while rumination varies across individuals. Simulated memory time is calculated for each individual and averaged over the study time horizon. We then calculate the average memory time for each category reported in Table 3 by gender. The table shows that, on average, it takes more time for girls to release a stressor (11.7 months) compared to boys (6.8 months) (first row in Table 3). This effect is driven by the higher gain for the Rumination reinforcing loop, R1, for girls. This loop is stronger in girls because they are estimated to have a higher baseline rumination tendency (θ_3_>0), so are more likely to see an increase in rumination, which sustains the stressful memories (i.e., increases memory time), leading to even more rumination. For those depressed individuals starting the study with high levels of rumination, both reinforcing loops remain active throughout the study period, and thus they all have long memory times regardless of gender (second row of Table 3). However, the stronger R1 loop can distinguish among depressed individuals starting the study with low levels of rumination: the lower strength of R1 increases the chance for boys to reduce rumination, and memory time, compared to girls (third row of Table 3). In sum, the gender effect in rumination means that girls may sustain stressful memories for longer (Fig 2), and as a result, have a higher risk of depression given the same external stressful events.

**Table 3:** Memory time by gender and level of rumination.

Participants	Girls	Boys	T-test p-value
All participants	11.7	6.8	0.00
Depressed individuals with high rumination (Rum_0_>mean rumination = 11.59)	20.4	19.5	0.10
Depressed individuals with low rumination (Rum_0_<mean rumination = 11.59)	9.3	6.5	0.00

### Trajectories of depressive symptoms by characteristics of participants

What are the implications of this estimated model for individual trajectories of depression? Individuals vary on many factors, including their initial state (i.e., level of active stressors, rumination, and depressive symptoms) and rate of exposure to stressors over time. An important question is how such individual-level variations manifest themselves in the long-term trajectories of depression. We can better appreciate the resulting variations using controlled simulation experiments. Therefore, we explore different potential trajectories by creating individual profiles varying across five parameters of the model: initial value of rumination, initial value of depressive symptoms, gender, prior stressors (i.e., the initial value of *past stressors kept alive* in Fig 1), and ongoing stressors (i.e., inflow of *past stressors kept alive* in Fig 1). A large value for prior stressors implies that a simulated participant has experienced many stressors in the six months prior to starting the simulation, while sizable ongoing stressors indicates that stressors continue to happen in someone’s life.

**Fig 3:**
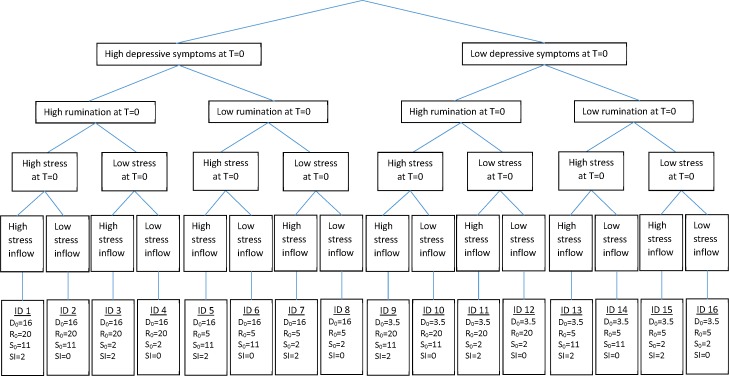
Sixteen categories of girl participants. D_0_, R_0_, S_0_, and SI represent initial depressive symptoms and rumination, prior stressors, and ongoing stressors, respectively.

We conducted a full-factorial simulation experiment varying the four factors for each gender at two levels: initial depressive symptoms, initial rumination, prior stressors, and ongoing stressors (i.e., flow of stressors). We ran the model for each of the resulting 16 boy/girl groups over 120 months. For brevity, we focus the discussion on the results for girls (Fig 3) and provide the counterparts for boys in S1 Fig Appendix. In each group, we used identical inputs (reported in the last row of Fig 3), instead of the actual individual data, in order to conduct fully controlled simulation experiments. The inputs defining the groups reported in Fig 3 were found by adding or subtracting one standard deviation to or from the empirical means of depression and rumination to determine the high or low levels of these variables. The same calculation using two standard deviations was used to find the high levels of stressors, and zero for low levels of stressors, to avoid negative stressor values. As a result, the first eight groups had high levels of depressive symptoms at the beginning of the simulation, and the rest had low initial depressive symptoms (Fig 3). Groups 1 to 4 and 9 to 12 had high initial rumination, while groups 5 to 8 and 13 to 16 had low levels of initial rumination. For instance, at the beginning of the simulation, a simulated person in group 1 had high levels of depressive symptoms and rumination. In addition, she had experienced multiple stressful events in the six months prior to the beginning of the simulation, and more stressors were happening in her life. A subject in group 2 had the same characteristics, but no more stressors were occurring in her life. As in the real world, simulated subjects also vary in the random environmental factors that influence their outcome trajectories. We captured these environmental variations as first-order auto-correlated noise terms operating on indicated rumination and depression. Thus, 2,500 subjects were simulated in each experimental group, differing only in the realization of random shocks (but not in the underlying distributions). The mean and the range enveloping 75% of simulated depression symptom trajectories over time are reported for each group. The same analysis was then repeated for boys (see S1 Fig Appendix).

**Fig 4:**
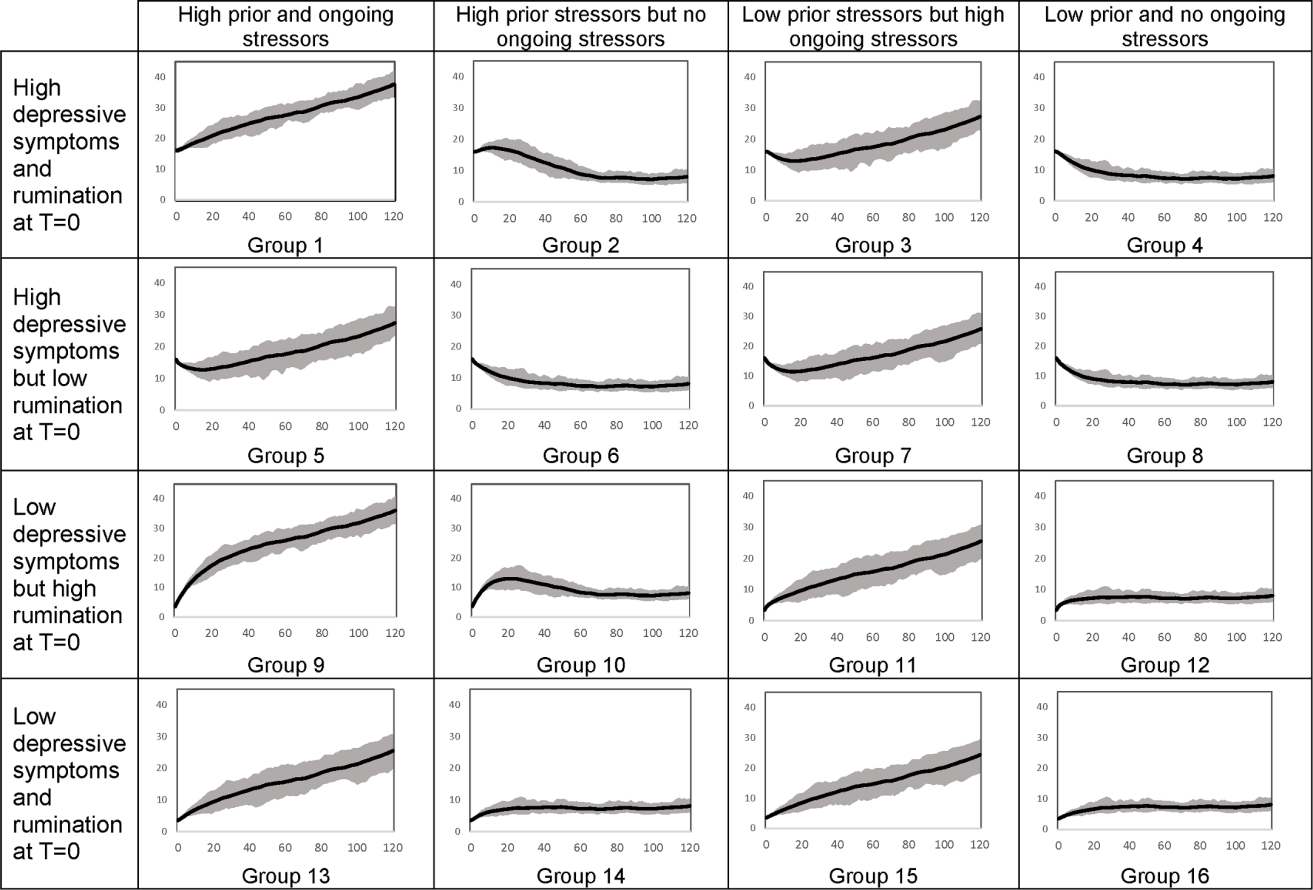
Simulated depressive symptoms over 120 months for 16 groups of girls with characteristics listed in Fig 3.

Fig 4 shows the mean and the 75% envelope of the simulated depressive symptoms over 120 months for each of the 16 profiles for girls. The 75% envelope shows the range containing all individuals with depressive symptoms between the 12.5 and 87.5 percentile within each group. Note that since we used the cut-off value reported in a study by Timbremont and Braet [47], a depression score above 16 depicts clinical depression. Comparing the simulated depressive symptoms in columns 1 with 2 and columns 3 with 4 indicates the critical role of ongoing stressors in the development of depression. Those with very high ongoing stressors (columns 1 and 3) experience increasing depressive symptoms, while those with no ongoing stressors (columns 2 and 4) have declining or stable depressive symptoms. For example, groups 1 and 2 experienced the same level of stressors six months prior to the start of the simulation, and they have the same initial depressive symptoms and rumination; but, unlike group 2, the stressors continue at a high rate for those in group 1. Thus, the depressive symptoms of group 2 increase slightly in the first months and then follow a declining trend, while the depressive symptoms of group 1 continue to increase during the entire simulation. Note that it is possible for a depressive episode to continue for a long time. A 30-year study of a large clinical sample found that 8% of patients had not recovered from a depressive episode after 10 years and 6% had still not recovered after 15 years [48]. However, the severity has fluctuations over time which do not appear in the simulations for two reasons. First, we used a constant flow for external stressors, whereas actual stressors are coming and going with much fluctuation. Moreover, in Fig 4, the fluctuations have been concealed because we are averaging over 2,500 individuals, but there are fluctuations in single simulations.

The initial increase in the depressive symptoms of group 2 is caused by previous stressors that are still present in the *past stressors kept alive* stock. This stock resembles a reservoir of stressful memories that is initially full. Despite the low inflow of stressful memories during simulation, the initially high levels of stressful memories keep the outflow from the reservoir low (R1), and can trigger more depressive symptoms (R2). However, once the *let it go* rate has depleted the stock enough, and in the absence of additional *ongoing stressors*, the *memory time* goes down, the *depressive symptoms* subside, and the *rumination* level declines as well. Unlike group 2, stressors continue to occur in the lives of individuals in group 1, thus the stressors accumulate in the stock of *past stressors kept alive* and the level of *rumination*, and subsequently *depression*, increases during the entire simulation. Due to the slow letting go of stressors, the simulated depressive symptoms of group 2 take more than ten years to reach equilibrium.

Prior stressors interact with the initial level of rumination to influence the initial trajectory of depressive symptoms. When both are high (groups 1, 2, 9 and 10), depressive symptoms initially grow because both the stressors stay around for longer (effect of loop R1) and the rumination increases depressive symptoms (effect of loop R2). In other words, higher initial rumination leads to lower *let it* go and keeps past stressors active for a longer time, which causes even higher *rumination* (Reinforcing loop R1 in Fig 1) and *depression* (Reinforcing loop R2 in Fig 1). In the simulations, the interaction between initial rumination and prior stressors is temporary, and final depressive symptoms are determined by ongoing stressors. For example, comparing group 10 (which is high on both factors) with group 12 or 14, the latter groups differ in initial trajectories but show similar final levels, largely determined by the ongoing stressors level (the same across all 3). However, long-term impacts of that interaction can be seen when high levels of initial stressors and rumination are accompanied by high ongoing stressors (groups 1 and 9); in these cases, very high final depressive symptoms emerge from the sustained slow *let it go* rates when depressive symptoms and rumination reinforce each other continuously. The difference between groups 9 and 1 is in the initial depressive symptoms (high in group 1, low in group 9), and is only salient in the short run (first 20 months), with limited impact on the longer-term trajectories.

**Fig 5:**
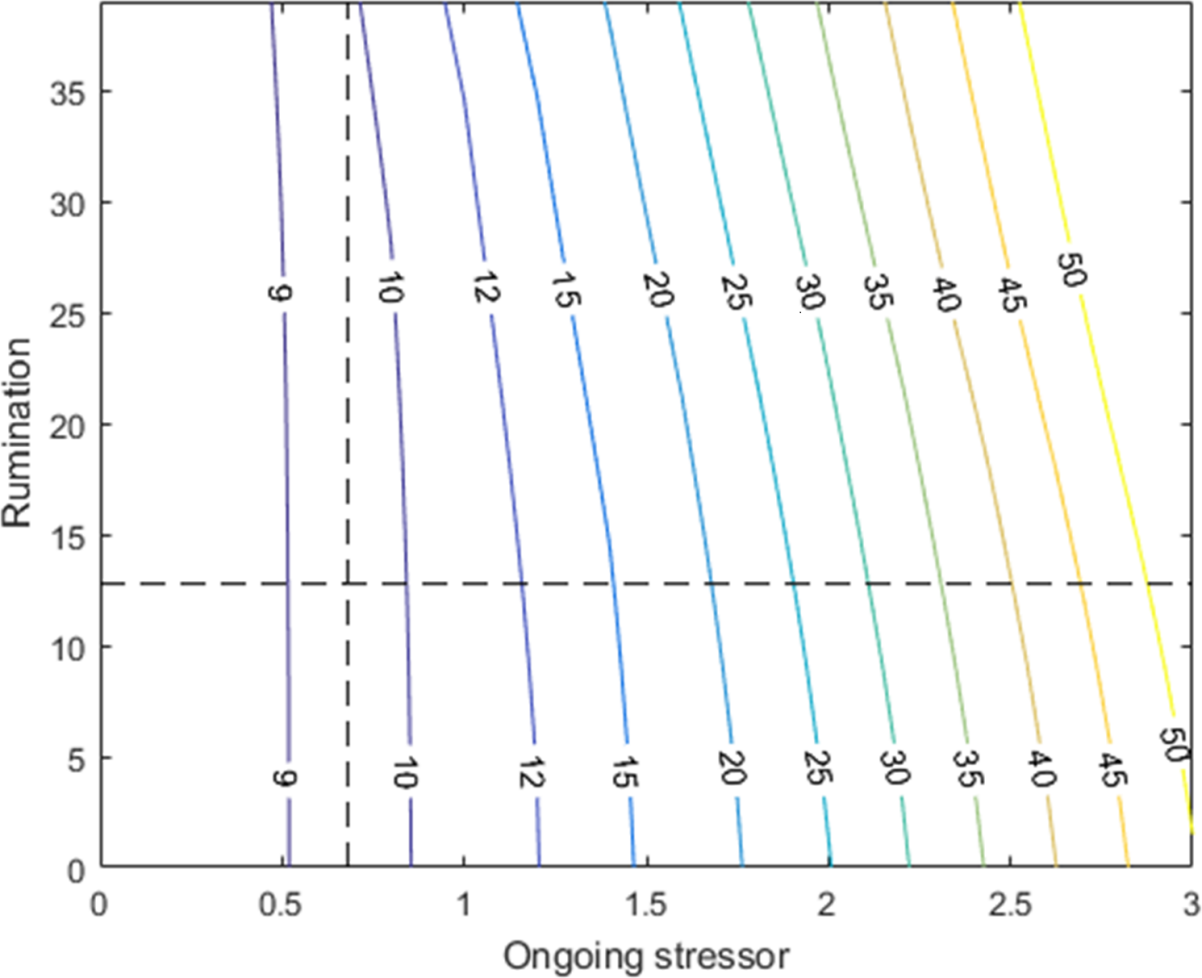
Depressive symptoms at time 120 by rumination and ongoing stressors for a simulated girl whose prior stressors and depressive symptoms are set at the mean.

Results shown in Fig 4 highlight the significant impact of ongoing stressors and rumination on depressive symptoms. However, the numbers used to generate the graphs in Fig 4 are just a few extreme combinations of the range we observed empirically. Thus, we ran a sensitivity analysis to investigate the depressive symptoms after 120 months of simulation as a function of stressor inflow (ongoing stressors) and initial rumination (Fig 5). The sensitivity analysis for girls was conducted by setting the initial level of depressive symptoms and prior stressors at their mean values, 9.98 and 4.97, respectively. The contour graph presented in Fig 5 illustrates mean depressive symptoms at time 120 for different combinations of ongoing stressors and initial rumination (depressive symptoms above 16 are considered to represent depression). The horizontal and vertical dashed lines represent the means for rumination and ongoing stressors. As expected, the depressive symptoms for the average individual are well below the depression threshold. Trajectories for boys are documented in S1 Fig Appendix.

## Discussion

Building on the response styles theory, this study investigated two of the potential feedback mechanisms underlying depression: in the first rumination increases the memory time for stressors and in the second it reinforces depressive symptoms. The proposed mechanisms were formalized in a simulation model that was estimated using data from a longitudinal study of depression and rumination among adolescents. The results indicate that rumination contributes to depression by keeping stressors ‘alive’, which in turn stimulate more rumination. Using a simulation method, we estimated the amount of time a person was activated by a stressor. On average, girls continued to ruminate about a stressor for 12 months, while it took about 7 months for boys to release a stressor. The same level of ongoing stressors creates less depressive symptoms in boys because girls are more likely to ruminate. The simulation model illustrated that the depressive symptoms of a non-depressed adolescent reached the depressive symptoms of an initially depressed person if they both had high levels of initial rumination, had experienced prior stressors, and were exposed to more stressors during the simulation. This indicates the importance of preventive care for non-depressed individuals with a ruminative style who are exposed to stress. Moreover, comparisons of individuals with the same characteristics, except for the inflow of stressors (ongoing stressors), demonstrated the critical role of this factor in the evolution of depression. Vulnerability to past events was moderated by the level of initial rumination.

Our findings reinforce and build on past studies in several ways. We found that girls ruminate significantly more than boys. A meta-analysis of the literature on rumination with a pooled sample of 14,321 individuals showed that rumination rate is significantly higher among adult females [49]. Similar to Michl and colleagues [5], we found that rumination mediated the relationship between stressful life events and depression. In line with findings reported by Nolen-Hoeksema and coauthors [15], we showed that rumination significantly increases depression, but we found only limited support for the effect of depression on rumination (there was a positive coefficient, but it was not statistically significant). However, in contrast to the joint estimation method we used, that study separately examined whether rumination in this period predicted depressive symptoms at the next time period and whether depressive symptoms in this period predicted rumination at a later time [15].

Besides reinforcing prior findings, the proposed model contributes to the literature in distinct ways. First, unlike previous studies that investigated reciprocal relationships between either stress and depression or rumination and depression, the proposed model simultaneously captures the reciprocal relationships between stress, rumination, and depression. Second, this is the first known study to examine one of the proposed pathways through which rumination contributes to depression by keeping stressors alive, and thus a person activated. Third, this model provides an integrated model of an individual’s endogenous response to exogenous stressors, and thus can be used in future examinations on the impact of multiple factors and/or interventions on trends of depressive symptoms. In other words, the model provides a simulation environment in which the effects of interventions can be estimated. Finally, this study offers the first individual-level system dynamics model of depression and is the first steppingstone to quantify the complex interactions among drivers of the disorder. The fully documented model, according to Rahmandad and Sterman [50]‘s guidelines, is available for other researchers to replicate and build on this work.

### Clinical implications

The present study highlights the importance of personalized prevention and intervention. Intervening to change the level of ongoing stressors, as well as the initial level of rumination and prior stressors, may generate diverse trends in depressive symptoms. Fast exacerbation of the symptoms for non-depressed individuals with a ruminative style who are facing stressors indicates that they may receive significant benefits from timely prevention. Since the time it takes to release a stressor is significantly different in boys and girls, gender should be one of the important determinants in tailoring treatment. By providing the full documentation of the model, we hope that practitioners can use our calibrated model for estimating the effects of interventions on depression in adolescents. For instance, one can input the initial level of depressive symptoms, initial rumination, gender, level of prior stressors, and current stressors (all measurable using standard instruments reported in this paper) of an adolescent into the model and then simulate the model for a certain time to predict the progression of depressive symptoms in the absence of interventions. Such benchmarks can then guide future assessments and adaptations of treatment plans. Also, a rumination intervention that reduces the memory time can be added to the model to examine the impact of the intervention on depressive symptoms and to determine the optimal timing and length of a treatment.

### Limitations

These findings should be interpreted in light of a few important limitations. First, many complex feedback mechanisms underlie depression [1]. Environmental factors as well as social, psychological, and biological factors determine the course of depression. This study only included two interdependent cognitive mechanisms and environmental stressors. However, by focusing on adolescents we attempt to rule out the existence of some biological mechanisms such as hippocampal atrophy, which is thought to take years to develop. Future research can expand upon this model to include additional mechanisms. Second, we relied on self-report questionnaire checklists which are susceptible to bias and recall failure. Specifically, life event checklist measures are increasingly considered less effective measures of stressful events. Comparisons of interview-based and self-report methods indicate that significant differences exist between the events captured by the two methods [51]. However, a review of studies on stress in children and adolescents showed that only 2% of the 500 studies used interview-based methods for measuring stress. The self-report life event checklists have remained the most common stress measure and research using secondary data have to rely on them. As discussed in the methods section and in S2 File Appendix, we addressed some of the limitations of the life events measure by estimating the stressors that contribute to rumination using the checklist. Another data limitation is that subjects may ruminate about stressors that were not measured. In addition, the rumination measure may also capture concepts such as worrying, which is more focused on the future. Some of the model parameters were estimated at the population level; if more data were available, individual estimates could be made, providing a more nuanced understanding of heterogeneity in various response functions among individuals. Finally, every model is a simplification of reality, and as such, its implications would only hold to the extent that the assumptions built into the model structure (e.g., the response styles theory and Ruscio et al.’s third pathway) are a good representation of the situation at hand. In addition to grounding the model in well-supported theoretical constructs, we built confidence in the structure of the model by checking the level of significance of the model parameters, nevertheless, estimation of feedback-rich models of depression is novel and more research is needed to assess, expand, and realize the full potential of these methods.

### Conclusion

The proposed model incorporates the current literature on one of the major mechanisms of depression. The results highlight the importance of individualized prevention and intervention for depression and support the idea that rumination contributes to depression by keeping stressors active.

## Supporting information

S1 File Developing and calibrating a model of depression. (DOCX)

S2 File Estimating the inflow of past stressors kept alive. (DOCX)

S1 Fig Trajectories for male participants. (DOCX)
