# Supplementary material for: Modeling and estimating the feedback mechanisms among depression, rumination, and stressors in adolescents
Source: PLoS One. 2018 Sep 27;13(9):e0204389. doi: 10.1371/journal.pone.0204389 (PMC6160072; doi:10.1371/journal.pone.0204389)
Supplement: S1 File — (DOCX) [file pone.0204389.s001.docx]

**Developing a model of depression**

We developed a system dynamics simulation model of depression that simultaneously captured the bidirectional relationships between depressive symptoms, rumination, and stressors at the individual level. Our model included two major feedback loops which are built on the mechanism proposed by [Ruscio, Gentes [1]](#_ENREF_1), depicted in reinforcing feedback loop 1 (Figure A, loop R1: ‘Rumination’), and the response style theory [[2](#_ENREF_2)], illustrated in reinforcing feedback loop 2 (Figure A, loop R2: ‘Symptom Exacerbation’). These two feedback loops are self-reinforcing such that an initial change in the loop comes back to cause a further change in the same direction—e.g., an initial increase will cause a further increase. For instance, the feedback loop R1 captures the idea that after experiencing a stressor, an individual prone to rumination spends time ruminating about the stressful events, keeping those stressors active, and thus increasing the chances of even more rumination. In other words, rumination intensifies sensitivity to stressors by keeping a person activated and the stressor “alive” [[1](#_ENREF_1)]. This leads to more *rumination* and *depressive symptoms*. The feedback loop R2 hypothesizes that more *rumination* leads to more *depressive symptoms* and a higher level of *depressive symptoms* causes even more *rumination* [[2](#_ENREF_2)]. It should be noted that the feedback loops R1 and R2 are interconnected (see Figure A). This is important because, similar with any other complex systems, the interaction between the feedback loops in the model gives rise to other potential dynamics—we discuss these dynamics further in the Results section.

As discussed, Figure A summarizes the key feedback loops (i.e., R1 and R2) in our model; however, a fully operational simulation model is needed in order to specify the mechanisms quantitatively. Next we will discuss the formulations used in the model shown in Figure A.

*Past stressors kept alive* and *depressive symptoms* influence *rumination* with a delay. As a result, current *rumination* is the first-order delay of *indicated rumination*. *Indicated rumination* is a linear function of *past stressors kept alive* [[3](#_ENREF_3)], *depressive symptoms* [[2](#_ENREF_2)], and *gender* [[4](#_ENREF_4)].

${indicated rumination}_{t}=(\theta_{1}+\theta_{2}\times{depressive symptoms}_{t}+\theta_{3}\times gender+\theta_{4}\times{past stressor kept alive}_{t})/(1-\theta_{5})$ (1)

Since it takes time for *rumination* to influence the *depressive symptoms*, the *depressive symptoms* is formulated as the first-order delay of *indicated depressive symptoms* which we assume is a function of *rumination*.

${indicated depressive symptoms}_{t}={(\theta}_{6}+\theta_{7}\times{rumination}_{t})/(1-\theta_{8})$ (2)

In addition, *indicated rumination* and *indicated depressive symptoms* are not deterministic and are affected by random events outside the model boundaries including various unmeasured environmental factors. Typically, there is some autocorrelation in how those chance events unfold which we capture using normally distributed first-order auto-correlated noise terms that are added to the *indicated rumination* and *indicated depressive symptoms*. $\theta_{10}$ and $\theta_{11}$ are the standard deviation of noise terms added to the *indicated rumination* and *indicated depressive symptoms* and $\theta_{12}$ is the correlation time.

The variable, *past stressors kept alive*, cannot be constructed directly from the Life Events Scale for Children [[5](#_ENREF_5)] because in this questionnaire participants reported stressful life events that they experienced in the past six months. Respondents might still ruminate about a stressor that happened more than six months ago or they might not ruminate about a stressor that happened less than six months ago. To overcome this limitation, we estimated *past stressors kept alive* and *ongoing stressors* from the stressful life events (see section 4 of this appendix). *Past stressors kept alive* is a stock variable—a ‘stock’ variable represents the sources of inertia and accumulations in a system and, mathematically, is represented as an integral. A stock variable is calculated by accumulating the difference between its inflow and outflow—a ‘flow’ variable (i.e., infolow or outflow) is the rate at which the ‘stock’ variable is chaging. Likewise *past stressors kept alive* is the accumulation of the difference between its inflows (i.e., *ongoing stressors*) and outflows (i.e., *let it go*: the process through which people let go of the stressor).

${past stressor kept alive}_{t}=\int_{t} \left[ ongoing stressor\left( s \right)-let it go\left( s \right) \right]ds+{past stressor kept alive}_{t0}$ (3)

${let it go}_{t}=\frac{{past stressor kept alive}_{t}}{{memory time}_{t}}$ (4)

${memory time}_{t}=\theta_{9}\times{rumination}_{t}$ (5)

Table A lists the 12 unknown parameters of the model; that is, the parameters that will be estimated. The formula of the model is listed in Table B. The model assumes individuals vary in their current state (i.e., the value of different stocks for the model of an individual) and the random shocks they are exposed to, but share the same underlying causal mechanisms (and thus the same parameters).

Figure A. The depression‑rumination conceptual model. Boxes depict stock (or state) variables and arrows with valves represent flows into/out of those stocks. Single-line arrows indicate causal relationships hypothesized among variables (the strength of which is estimated below). A stock variable is the accumulation of the difference between its inflows and outflows and, mathematically, is represented as an integral.

Table A. Unknown Parameters in the Model

| **Unknown Parameters (θ)** | **Unit** |
| --- | --- |
| Rumination constant (θ_1_) | RumScore |
| Effect of depressive symptoms on rumination (θ_2_) | RumScore/DepScore |
| Gender coefficient (θ_3_) | RumScore |
| Effect of stressors on rumination (θ_4_) | RumScore/Disruption |
| Rumination coefficient (θ_5_) | Dmnl* |
| Depression constant (θ_6_) | DepScore |
| Effect of rumination on depressive symptoms (θ_7_) | DepScore/RumScore |
| Depression Coefficient (θ_8_) | Dmnl |
| Effect of rumination on memory time (θ_9_) | 1/RumScore |
| Standard deviation of rumination noise (θ_10_) | Dmnl |
| Standard deviation of depression noise (θ_11_) | Dmnl |
| Correlation time (θ_12_) | Month |

*Dimensionless

**Table B: The MDD-Rumination Model**

| **Formulations** | **Units** |
| --- | --- |
| *Adjusted Stressor0=Stressor0*Memory Time/Measurement time* | *engram* |
| *Change in PinkNoise MDD=(WhiteNoise MDD-PinkNoise MDD) / Ɵ12* | *DepScore/Month* |
| *Change in PinkNoise Rum= (WhiteNoise Rum-PinkNoise Rum) / Ɵ12* | *RumScore/Month* |
| *Effect of Rumination on memory time=1.4741* | *1/RumScore* |
| *Fraction of Stimuli Negatively Perceived= 1* | *Disruption/engram* |
| *Gender=GET XLS CONSTANTS( 'AdolescentData.xlsx' , 'Sheet1' , 'B2')* | *Dmnl* |
| *Indicated MDD=max(0, (Ɵ6+Ɵ7*Rumination) /(1-Ɵ8) + PinkNoise MDD*MDDSwitch)* | *DepScore* |
| *Indicated Rumination=max(0, (Ɵ1+Ɵ2*Depressive symptoms+Ɵ3*Gender+Ɵ4*Perceived Negative Stimuli)/(1-Ɵ5) +PinkNoise Rum*RumSwitch)* | *RumScore* |
| *Let it go=(past stressors kept alive)/Memory Time* | *engram/Month* |
| *Depressive symptoms=*  *SMOOTHI(Indicated MDD, Minimum Adjustment time/(1-Ɵ8) , MDD0)* | *DepScore* |
| *MDD0=GET XLS CONSTANTS( 'AdolescentData.xlsx' , 'Sheet1' , 'B4')* | *DepScore* |
| *MDDResidualMean=0* | *DepScore* |
| *MDDSwitch=0* | *Dmnl* |
| *Measurement time=6* | *Month* |
| *Memory Time=Minimum Memory Time*Rumination* Ɵ9* | *Month* |
| *Minimum Adjustment time=1* | *Month* |
| *Minimum Memory Time=1* | *Month* |
| *NoiseSeedMDD=3* | *Dmnl* |
| *NoiseSeedRum=2* | *Dmnl* |
| *Perceived Negative Stimuli=*  *past stressors kept alive *Fraction of Stimuli Negatively Perceived* | *Disruption* |
| $PinkNoise MDD(t)= PinkNoise MDD(0)+ \int_{0}^{t} (Change in PinkNoise MDD)dt$*; PinkNoise MDD(0)=0* | *DepScore* |
| $PinkNoise Rum(t)= PinkNoise Rum(0)+ \int_{0}^{t} (Change in PinkNoise Rum)dt$*; PinkNoise Rum(0)=0* | *RumScore* |
| *Rum0= GET XLS CONSTANTS( 'AdolescentData.xlsx' , 'Sheet1' , 'B3')* | *RumScore* |
| *Rumination=SMOOTHI(Indicated Rumination, Minimum Adjustment time/(1-Ɵ5) , Rum0 )* | *RumScore* |
| *RumResidualMean=0* | *RumScore* |
| *RumSwitch=0* | *Dmnl* |
| *Ongoing stressors=GET XLS CONSTANTS( 'AdolescentData.xlsx' , 'Sheet1' , 'B6')* | *engram/Month* |
| $past stressors kept alive\left( t \right)= past stressors kept alive\left( 0 \right)+ \int_{0}^{t} \left( ongoing stressors-Let it go \right)dt$*; past stressors kept alive (0)=Adjusted Stressor0* | *engram* |
| Stressor0=GET XLS CONSTANTS( 'AdolescentData.xlsx' , 'Sheet1' , 'B5') | engram |
| TIME STEP = 0.03125 | Month |
| *WhiteNoise MDD=RANDOM NORMAL( -10 , 10 , MDDResidualMean , 1 , NoiseSeedMDD )*(( Ɵ11^2)*(2-(TIME STEP/ Ɵ12)) /(TIME STEP/ Ɵ12))^0.5* | *DepScore* |
| *The formulation of white noise and pink noise are taken from Sterman (2000).* |  |
| *WhiteNoise Rum=RANDOM NORMAL( -10 , 10 , RumResidualMean , 1 , NoiseSeedRum )*(( Ɵ10^2)*(2-(TIME STEP/ Ɵ12)) /(TIME STEP/ Ɵ12))^0.5* | *RumScore* |
| *Ɵ1=-1.2504* | *RumScore* |
| *Ɵ2=0.4236* | *RumScore/DepScore* |
| *Ɵ3=2.5152* | *RumScore* |
| *Ɵ4=0.2518* | *RumScore/Disruption* |
| *Ɵ5=0.1639* | *Dmnl* |
| *Ɵ6=0.3730* | *DepScore* |
| *Ɵ7=0.0699* | *DepScore/RumScore* |
| *Ɵ8=0.8894* | *Dmnl* |
| *Ɵ9=1.4741* | *1/ RumScore* |
| *Ɵ10=7.8735* | *Dmnl* |
| *Ɵ11=0.0002* | *Dmnl* |
| *Ɵ12=1.6008* | *Month* |

**Calibration of the model using indirect inference method**

The unknown parameters of the model are estimated by using the indirect inference method [[6](#_ENREF_6), [7](#_ENREF_7)]. This method is useful for estimating complex models with intractable likelihood function or dynamic models when there are only a few data points over time. In this method, the unknown parameters of a model (θ) are estimated by matching different statistics of the empirical data to simulated data generated by simulating the model of interest. This method has been applied in various fields for estimating different types of models including nonlinear ecological dynamic system [[8](#_ENREF_8)], dynamic models with intractable likelihood function [[9](#_ENREF_9)], continuous time models [[10](#_ENREF_10)], and stochastic volatility models [[11](#_ENREF_11)]. [Hosseinichimeh, Rahmandad [12]](#_ENREF_12) discuss the implementation of the indirect inference for dynamic models and specifically show how the proposed model in Figure A is estimated. The basic intuition behind indirect inference is to create a set of auxiliary estimation models (e.g., linear regressions specifying various relationships among rumination, depression, and stressors) and estimate them first by using the empirical data. Then simulate the core model (in this case, the model specified in Figure A) and, using optimization techniques, find parameters of the core model such that the coefficients estimated in the first step match the coefficients of the auxiliary models found by using the simulated data from the core model. The summary of the estimation processes, the indirect inference method, follows:

Consider a general dynamic model with stock (state) variables z, dynamics of which are described as $\frac{\mathrm{dz}}{dt}=f(\theta_{1},z,{u,ԑ}_{1})$ and a set of exogenous variables, *u*, and observable variables, x, which are a function of z:

$x=g(\theta_{2},z,{u,ԑ}_{2})$ (1)

Here function *f* describes the dynamics of the system and function *g* the measurement process and structure of both of these functions are assumed to be known. A vector of random errors with a known distribution^[[1]](#footnote-1)^ ($\varepsilon=\bigcup(\varepsilon_{2},\varepsilon_{1})$) adds uncertainty to the dynamics and measurements. Finally, a set of parameters, $\theta=\bigcup(\theta_{2},\theta_{1})$, is unknown and the goal of the estimation process is to find these parameters. Note that the model and measurement functions may apply to a single case or multiple units of the phenomenon of interest. For example, a panel dataset includes measures on dynamics of several parallel units (e.g., people, firms, or countries) over time. Figure B summarizes the steps to estimate the model parameters (θ) by using the indirect inference method.

First, suitable statistics of empirical data, *x*, are generated. Suitable statistics include coefficients of an auxiliary model (e.g., a regression that estimates some elements of x based on other elements or lagged values) or they can be any statistics of a dataset such as mean and standard deviation [[8](#_ENREF_8)]. We call them empirical-auxiliary statistics (S_EmpAux_). Second, the corresponding simulated statistics, simulated-auxiliary statistics (S_SimAux_), are calculated and estimated. For a given value of θ, the model of interest (SD model) is simulated H times by using H different streams of noise over time, ԑ_t_ (=$ԑ_{1}^{h}$,…$,ԑ_{T}^{h})$, h=1,…H. As a result, H number of *x* are generated. Then, S_SimAux_ is estimated for each *x*. Third, the average of these estimators are found ($\frac{1}{H}\sum_{h=1}^{H} {S_{SimAux}}^{h}$) and θ is changed to minimize the difference between the empirical-auxiliary statistics and the average of simulated-auxiliary statistics [[6](#_ENREF_6)]. For a detailed description of the calibration process of this model see [Hosseinichimeh, Rahmandad [13]](#_ENREF_13).

θ

***Optimization Iterations until*** $\boldsymbol{(S-}\bar{\boldsymbol{S}}\boldsymbol{)W(S-}\bar{\boldsymbol{S}}\boldsymbol{)}$ ***is minimized***

Simulated Data

Model of interest

(SD model)

Empirical Data

*S=S_1_, S_2_…, S_k_*

$\bar{S}$ =$\bar{S_{1}}(\theta)$, $\bar{S_{2}}(\theta)$,…, $\bar{S_{k}}(\theta)$

Minimize ($S-\bar{S})W(S-\bar{S})$

$$\hat{\theta}_{IndirectInference}$$

Conversion of data to statistics

Figure B. Required steps for estimating parameters of a dynamic model

**References**

1. Ruscio, A.M., et al., *Rumination predicts heightened responding to stressful life events in major depressive disorder and generalized anxiety disorder.* Journal of Abnormal Psychology, 2015. **124**(1): p. 17-26.

2. Nolen-Hoeksema, S., et al., *Reciprocal relations between rumination and bulimic, substance abuse, and depressive symptoms in female adolescents.* Journal of Abnormal Psychology, 2007. **116**(1): p. 198-207.

3. Michl, L.C., et al., *Rumination as a mechanism linking stressful life events to symptoms of depression and anxiety: Longitudinal evidence in early adolescents and adults.* Journal of Abnormal Psychology, 2013. **122**(2): p. 339-352.

4. Nolen-Hoeksema, S., J. Larson, and C. Grayson, *Explaining the gender difference in depressive symptoms.* Journal of Personality and Social Psychology, 1999. **77**(5): p. 1061-1072.

5. Coddington, R.D., *The significance of life events as etiologic factors in the diseases of children: I—A survey of professional workers.* Journal of Psychosomatic Research, 1972. **16**(1): p. 7-18.

6. Gourieroux, C., A. Monfort, and E. Renault, *Indirect Inference.* J. of App. Econ., 1993. **8**: p. S85-S118.

7. Smith, A.A., *Estimating nonlinear time-series models using simulated vector autoregressions.* Journal of Applied Econometrics, 1993. **8**(S1): p. S63-S84.

8. Wood, S.N., *Statistical inference for noisy nonlinear ecological dynamic systems.* Nature, 2010. **466**(7310): p. 1102-1104.

9. Gouriéroux, C., P.C. Phillips, and J. Yu, *Indirect inference for dynamic panel models.* Journal of Econometrics, 2010. **157**(1): p. 68-77.

10. Monfort, A., *A Reappraisal of Misspecified Econometric Models.* Econometric Theory, 1996. **12**(4): p. 597-619.

11. Monfardini, C., *Estimating Stochastic Volatility Models Through Indirect Inference.* The Econometrics Journal, 1998. **1**(1): p. 113-128.

12. Hosseinichimeh, N., et al., *Using indirect inference method for parameter estimation in dynamic models, revise and resubmit from System Dynamics Review*. 2016.

13. Hosseinichimeh, N., et al., *Estimating System Dynamics Models Using Indirect Inference.* minor revision from System Dynamics Review, 2016.

1. The distribution of the ԑ does not need to be known. ԑ can be a function of a white noise with a known distribution and an unknown parameter of the model of interest (θ) (Gourieroux et al. 1993). Moreover, if there is uncertainty in the initial conditions of stock variables, that uncertainty could be incorporated into the ԑ. [↑](#footnote-ref-1)
