# Supplementary material for: Modeling and estimating the feedback mechanisms among depression, rumination, and stressors in adolescents
Source: PLoS One. 2018 Sep 27;13(9):e0204389. doi: 10.1371/journal.pone.0204389 (PMC6160072; doi:10.1371/journal.pone.0204389)
Supplement: S2 File — (DOCX) [file pone.0204389.s002.docx]

**Estimation of the inflow of “past stressors kept alive” from the reported stressful life events**

In the Life Events Scale for Children questionnaire [[1](#_ENREF_1)], participants report the stressful life events that they experienced in the past six months. The problem with this measure is that a participant might not ruminate about a stressful event reported in the questionnaire which happened less than 6 months ago. Also, we do not know if there were some other events experienced more than 6 months ago that still contribute to current rumination. To overcome these data limitations, we constructed a new variable, *past stressors kept alive*, from the *reported stressful life events* measured by Life Events Scale for Children. The former captures the stressors that still contribute to rumination and the latter is what individuals have reported in the questionnaire. These two variables are the accumulation of stressors and they have the same inflow (i.e., they are both increased by the same inflow of stressors) while they have different outflows.^[[1]](#footnote-1)^ The main idea is to estimate the inflow of “*reported stressful life events*” by using the data from the Life Event Questionnaire and then use that inflow for the new variable “*past stressor kept alive*”. The process occurs as follows.

Consider a bathtub shown in Figure 3 representing the “*reported stressful life events*”. As individuals experience new stressors (i.e., ongoing stressors in the following figure), the level of experienced stressors increases in that stock. Stressors are written off after six months (in the questionnaire, individuals are asked to report only stressors that they have experienced in the past six months). Thus, on average, stressors stay in that bathtub for six months. In other words, the outflow is formulated as “*reported stressful life events*” divided by six. Equation 1 and 2 show the formula for the stock of “*reported stressful life events” and its outflow respectively.*

$reported stressful life events\left( t \right)=reported stressful life events\left( t0 \right)+\int_{t0}^{t} [ongoing stressors(s)-deleting stressors(s)]ds$ *(1)*

$deleting stresors=\frac{reported stressful life events}{6}$ (2)

“*reported stressful life events” is derived from the* Life Event Questionnaire*. The outflow, deleting stressors, is also known (it is reported stressful life event divided by six). The inflow, ongoing stressors, is estimated by simulating equation 1 and 2 for all individuals in our sample, and minimizing the squared of the difference between simulated* “*reported stressful life events” and the empirical* “*reported stressful life events”.* The estimated inflow, *ongoing stressors*, will be used in the formulation of “*past stressors kept alive*” described in the manuscript. Mean and standard deviation of estimated *ongoing stressors* are 0.68 and 0.78 respectively.


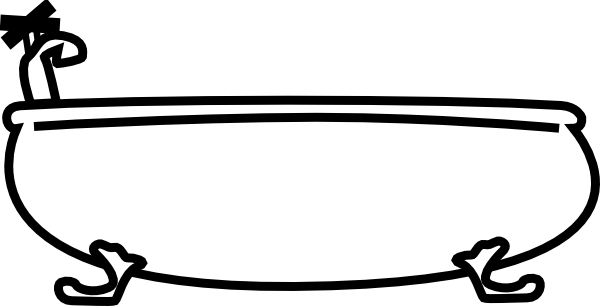


Stock of reported stressful life event

Ongoing stressors

Deleting stressors after 6 months

Figure 3. Bathtub example

**References**

1. Coddington, R.D., *The significance of life events as etiologic factors in the diseases of children: I—A survey of professional workers.* Journal of Psychosomatic Research, 1972. **16**(1): p. 7-18.

1. The outflow of the “past stressor kept alive” depends on the level of rumination and stressors while the outflow of reported stressful life events captures the leave of stressors after six months. [↑](#footnote-ref-1)
