# Supplementary material for: Modeling and estimating the feedback mechanisms among depression, rumination, and stressors in adolescents
Source: PLoS One. 2018 Sep 27;13(9):e0204389. doi: 10.1371/journal.pone.0204389 (PMC6160072; doi:10.1371/journal.pone.0204389)
Supplement: S1 Fig — (DOCX) [file pone.0204389.s003.docx]

|  | High prior and ongoing stressors | High prior stressors but no ongoing stressors | Low prior stressors but high ongoing stressors | Low prior and ongoing stressors |
| --- | --- | --- | --- | --- |
| High depressive symptoms and rumination at T=0 |  |  |  |  |
|  | Group 1 | Group 2 | Group 3 | Group 4 |
| High depressive symptoms but low rumination at T=0 |  |  |  |  |
|  | Group 5 | Group 6 | Group 7 | Group 8 |
| Low depressive symptoms but high rumination at T=0 |  |  |  |  |
|  | Group 9 | Group 10 | Group 11 | Group 12 |
| Low depressive symptoms and rumination at T=0 |  |  |  |  |
|  | Group 13 | Group 14 | Group 15 | Group 16 |

Mean and the 75% envelope of the simulated depressive symptoms for 16 male participant groups over 120 months. Using the same procedure as for girls, scores of 15/3, 17/3, 12/1.4, and 2/0 are assigned for high/low levels of depression, rumination, prior stressors and ongoing stressors, respectively, for boys.
